# Supplementary material for: Variation of the Tegmen and Cercus in Sinopodisma rostellocerca (Orthoptera: Acrididae: Melanoplinae) with Proposal of a New Synonym
Source: Insects. 2024 Jul 12;15(7):526. doi: 10.3390/insects15070526 (PMC11276630; doi:10.3390/insects15070526)
Supplement: Supplementary file 1 [file insects-15-00526-s001.zip › insects-3027168-supplementary.pdf]

Table S1. F and p value resulted from variance analysis of body length of male among populations of *Sinopodisma rostellocera* and *S. hengshanica*.

	Sr_hnxx	Sr_hngwj	Sr_hnjmx	Sr_hnyms	Sr_hndpl	Sr_gxmes	Sr_gxgc	Sr_gxhp	Sr_gxys	Sh_hnhs
Sr_hnxx	0	0.091816	0.087467	29.808183	3.617501	2.260983	0.043879	0.699715	4.184315	1.856132
Sr_hngwj	0.763139	0	0.002382	44.700209	3.367911	2.49435	0.167823	1.251624	4.977821	1.65173
Sr_hnjmx	0.769121	0.961265	0	52.025831	13.431038	2.43083	0.364145	1.714972	24.271492	7.081028
Sr_hnyms	0.000002	0	0	0	28.207181	33.506315	10.210541	4.063488	0.260839	18.596114
Sr_hndpl	0.070981	0.074988	0.001444	0.000013	0	0.173309	3.432985	2.381915	21.282242	0.14903
Sr_gxmes	0.142181	0.120963	0.12851	0.000001	0.682109	0	1.022068	1.932484	2.899823	0.029322
Sr_gxgc	0.836006	0.684481	0.552385	0.003445	0.106324	0.324731	0	0.17512	2.981038	1.860877
Sr_gxhp	0.411874	0.270652	0.203854	0.053507	0.166656	0.180559	0.686609	0	0.621072	1.369979
Sr_gxys	0.055699	0.032808	0.000109	0.614211	0.019163	0.109214	0.159321	0.474727	0	17.222105
Sh_hnhs	0.188219	0.20741	0.015002	0.000206	0.715344	0.86606	0.221475	0.286196	0.053452	0

Note: F value is in upper-right triangular region and p value is in lower-left triangular region.

Table S2. F and p value resulted from variance analysis of body length of female among populations of *Sinopodisma rostellocera* and *S. hengshanica*.

	Sr_hnxx	Sr_hngwj	Sr_hnjmx	Sr_hnyms	Sr_hnwyj	Sr_gxmes	Sr_gxgc	Sr_gxhp	Sr_gxys	Sh_hnhs
Sr_hnxx	0	0.249829	0.988009	20.58164	7.800029	2.357214	0.939872	0.000873	0.039158	1.401173
Sr_hngwj	0.618231	0	3.535299	36.837278	17.875654	6.648744	2.490393	0.069046	0.027024	2.132017
Sr_hnjmx	0.324358	0.063107	0	25.818037	5.141693	0.449675	0.299272	0.413656	0.213217	2.820356
Sr_hnyms	0.000031	0	0.000007	0	24.440502	24.459545	7.557144	7.6109	0.911332	1.239164
Sr_hnwyj	0.008136	0.000065	0.031269	0.000043	0	3.035139	1.573648	3.366476	4.695866	12.585714
Sr_gxmes	0.131271	0.011624	0.506545	0.000019	0.09854	0	0.032757	0.981496	0.44133	3.933967
Sr_gxgc	0.33878	0.118809	0.589004	0.01143	0.256332	0.858649	0	0.404896	0.458799	2.516846
Sr_gxhp	0.976564	0.793389	0.524434	0.009646	0.087871	0.331714	0.53652	0	0.014431	0.557846
Sr_gxys	0.844315	0.869885	0.648416	0.350622	0.096133	0.517274	0.568034	0.90676	0	0.145679
Sh_hnhs	0.244077	0.148427	0.104613	0.276658	0.009373	0.063712	0.173495	0.468423	0.728129	0

Note: F value is in upper-right triangular region and p value is in lower-left triangular region.

Table S3. F and p value resulted from variance analysis of pronotum length of male among populations of *Sinopodisma rostellocera* and *S. hengshanica*.

	Sr_hnxx	Sr_hngwj	Sr_hnjmx	Sr_hnyms	Sr_hndpl	Sr_gxmes	Sr_gxgc	Sr_gxhp	Sr_gxys	Sh_hnhs
Sr_hnxx	0	2.947492	0.00018	77.404444	8.551696	3.145863	0.8098	2.167282	0.081198	1.418202
Sr_hngwj	0.092201	0	2.994113	74.465789	16.610424	12.03938	0.039492	0.521733	0.62909	4.422936
Sr_hnjmx	0.98936	0.089736	0	77.46077	8.457458	3.10457	0.819357	2.181315	0.078314	1.392361
Sr_hnyms	0	0	0	0	59.167299	74.716152	11.851364	11.350382	7.868148	30.241822
Sr_hndpl	0.008105	0.000251	0.008408	0	0	0.909696	3.309764	6.959619	2.024849	2.904219
Sr_gxmes	0.085349	0.001126	0.087335	0	0.352829	0	2.381597	3.970648	0.089668	0.006009
Sr_gxgc	0.377925	0.843596	0.375175	0.00183	0.111687	0.139264	0	0.042357	0.130907	0.939113
Sr_gxhp	0.155138	0.47477	0.153868	0.002212	0.033515	0.060864	0.842081	0	0.391296	2.39559
Sr_gxys	0.778933	0.433531	0.782786	0.009814	0.249909	0.768709	0.735797	0.565537	0	1
Sh_hnhs	0.247642	0.042936	0.251852	0.000009	0.149072	0.939115	0.369931	0.172644	0.42265	0

Note: F value is in upper-right triangular region and p value is in lower-left triangular region.

Table S4. F and p value resulted from variance analysis of pronotum length of female among populations of *Sinopodisma rostellocera* and *S. hengshanica*.

	Sr_hnxx	Sr_hngwj	Sr_hnjmx	Sr_hnyms	Sr_hnwyj	Sr_gxmes	Sr_gxgc	Sr_gxhp	Sr_gxys	Sh_hnhs
Sr_hnxx	0	0.00124	3.19638	24.786972	12.724935	22.944909	0.164131	1.263565	0.044417	3.761297
Sr_hngwj	0.971978	0	3.58826	32.769661	9.178711	25.411972	0.105544	1.27321	0.028391	2.968733
Sr_hnjmx	0.079026	0.061199	0	25.238361	2.59883	6.446012	0.148607	0.021858	0.056336	0.426534
Sr_hnyms	0.000007	0	0.000008	0	15.899567	36.589512	3.587241	11.702896	1.162265	8.395023
Sr_hnwyj	0.000996	0.003345	0.118159	0.000512	0	0.067629	7.8522	1.824861	2.422121	0.44606
Sr_gxmes	0.000016	0.000003	0.015337	0.000001	0.797772	0	2.437181	3.807783	0.822583	0.634911
Sr_gxgc	0.687779	0.746192	0.703006	0.070869	0.031075	0.138049	0	0.050328	0.032258	0.724533
Sr_gxhp	0.267073	0.262456	0.883339	0.00177	0.198166	0.062781	0.826267	0	0.019507	0.331186
Sr_gxys	0.834337	0.866666	0.814398	0.293225	0.194618	0.379772	0.874012	0.891697	0	0.214515
Sh_hnhs	0.060105	0.089009	0.519218	0.007908	0.525612	0.436547	0.433524	0.574788	0.674782	0

Note: F value is in upper-right triangular region and p value is in lower-left triangular region.

Table S5. F and p value resulted from variance analysis of tegmen length of male among populations of *Sinopodisma rostellocera* and *S. hengshanica*.

	Sr_hnxx	Sr_hngwj	Sr_hnjmx	Sr_hnyms	Sr_hndpl	Sr_gxmes	Sr_gxgc	Sr_gxhp	Sr_gxys	Sh_hnhs
Sr_hnxx	0	1.526029	2.595202	7.120742	23.505213	0.266949	0.410283	0.147442	19.71122	1.709144
Sr_hngwj	0.222483	0	6.576345	2.309514	19.431304	2.010537	1.291782	0.864814	5.804879	1.867301
Sr_hnjmx	0.115922	0.013385	0	13.89618	10.043091	0.283918	0.070962	0.26107	11.973652	0.034824
Sr_hnyms	0.010782	0.134211	0.000572	0	22.67135	5.491003	3.257167	2.671505	3.533648	3.742657
Sr_hndpl	0.000086	0.000094	0.004623	0.000058	0	5.894577	3.672098	4.620509	8.15777	2.926011
Sr_gxmes	0.608831	0.162806	0.597715	0.024307	0.025899	0	0.010861	0.002792	3.982978	0.131972
Sr_gxgc	0.528442	0.263229	0.792422	0.081878	0.09686	0.91809	0	0.017981	3.776661	0.069516
Sr_gxhp	0.704678	0.358589	0.614476	0.113354	0.068661	0.958408	0.896641	0	4.371991	0.186696
Sr_gxys	0.000316	0.02191	0.002792	0.072329	0.06478	0.064451	0.123898	0.104721	0	17.077009
Sh_hnhs	0.205927	0.180754	0.853845	0.063991	0.147851	0.720874	0.800864	0.680777	0.05387	0

Note: F value is in upper-right triangular region and p value is in lower-left triangular region.

Table S6. F and p value resulted from variance analysis of tegmen length of female among populations of *Sinopodisma rostellocera* and *S. hengshanica*.

	Sr_hnxx	Sr_hngwj	Sr_hnjmx	Sr_hnyms	Sr_hnwyj	Sr_gxmes	Sr_gxgc	Sr_gxhp	Sr_gxys	Sh_hnhs
Sr_hnxx	0	3.083973	1.296896	14.729189	0.00461	22.590938	0.004287	0.346478	12.771364	0.038864
Sr_hngwj	0.081955	0	0.063723	33.597954	0.713825	15.226378	0.298774	2.882105	14.737205	0.200325
Sr_hnjmx	0.259463	0.801245	0	17.901282	0.430977	11.53247	0.174347	1.588021	12.780181	0.08242
Sr_hnyms	0.000323	0	0.000113	0	3.261697	40.243298	2.313932	3.855035	2.931249	2.69941
Sr_hnwyj	0.946222	0.400829	0.516869	0.082971	0	7.54629	0.012705	0.080486	12.525316	0.021016
Sr_gxmes	0.000019	0.000189	0.001615	0	0.013255	0	4.574314	12.904633	18.86503	3.403392
Sr_gxgc	0.94816	0.586296	0.679705	0.14185	0.913932	0.048223	0	0.101047	19.364788	0.003732
Sr_gxhp	0.559124	0.093361	0.21619	0.058621	0.780792	0.001465	0.75604	0	5.951854	0.140837
Sr_gxys	0.001078	0.000263	0.00153	0.10161	0.024033	0.000675	0.047956	0.03487	0	2.555875
Sh_hnhs	0.844798	0.655748	0.776236	0.113424	0.888821	0.082558	0.953653	0.713502	0.208181	0

Note: F value is in upper-right triangular region and p value is in lower-left triangular region.

Table S7. F and p value resulted from variance analysis of hind femur length of male among populations of *Sinopodisma rostellocera* and *S. hengshanica*.

	Sr_hnxx	Sr_hngwj	Sr_hnjmx	Sr_hnyms	Sr_hndpl	Sr_gxmes	Sr_gxgc	Sr_gxhp	Sr_gxys	Sh_hnhs
Sr_hnxx	0	0.003702	0.037363	39.803682	8.910769	4.455003	0.199153	0.556237	0.309065	6.635451
Sr_hngwj	0.951727	0	0.075353	54.157809	8.918597	6.278654	0.196412	0.582131	0.283098	6.701421
Sr_hnjmx	0.847815	0.784828	0	44.040638	9.626356	4.086825	0.340778	0.809357	0.444167	7.154592
Sr_hnyms	0	0	0	0	33.527173	51.691477	9.557955	8.040364	1.642598	25.029388
Sr_hndpl	0.007058	0.005127	0.00539	0.000004	0	0.728568	5.150874	6.214209	23.107537	0.000545
Sr_gxmes	0.042461	0.015741	0.05139	0	0.404564	0	2.229023	2.935567	0.913852	0.55351
Sr_gxgc	0.659767	0.660285	0.565318	0.004473	0.057515	0.15186	0	0.030349	0.037301	3.662392
Sr_gxhp	0.463676	0.450449	0.378053	0.008402	0.041425	0.102918	0.866029	0	0.008699	4.413704
Sr_gxys	0.585099	0.598353	0.51357	0.212214	0.01714	0.354249	0.856263	0.930175	0	9.3025
Sh_hnhs	0.018041	0.014075	0.014558	0.000033	0.982281	0.467047	0.104167	0.08038	0.09278	0

Note: F value is in upper-right triangular region and p value is in lower-left triangular region.

Table S8. F and p value resulted from variance analysis of hind femur length of female among populations of *Sinopodisma rostellocera* and *S. hengshanica*.

	Sr_hnxx	Sr_hngwj	Sr_hnjmx	Sr_hnyms	Sr_hnwyj	Sr_gxmes	Sr_gxgc	Sr_gxhp	Sr_gxys	Sh_hnhs
Sr_hnxx	0	2.189775	1.000462	25.412184	0.254801	5.404749	0.175111	0.421321	0.515533	1.273996
Sr_hngwj	0.141896	0	6.569269	27.889349	1.531591	14.304454	0.006839	0.027426	0.221464	0.368826
Sr_hnjmx	0.321353	0.011929	0	30.749587	6.40E-05	2.957475	1.17735	2.02222	1.565964	4.38317
Sr_hnyms	0.000005	0.000001	0.000001	0	7.25957	28.283308	2.543475	7.398591	0.375738	2.035147
Sr_hnwyj	0.61663	0.219684	0.993693	0.012419	0	0.990024	1.317496	0.624397	1.587314	4.714832
Sr_gxmes	0.024358	0.000287	0.093618	0.000006	0.332925	0	2.659338	4.760811	1.973299	6.33507
Sr_gxgc	0.678095	0.934315	0.287855	0.124401	0.294739	0.122468	0	0.000101	22.549363	4.197641
Sr_gxhp	0.519653	0.868873	0.164122	0.010602	0.442597	0.039138	0.992136	0	0.145061	0.278111
Sr_gxys	0.477658	0.63935	0.222855	0.546475	0.276206	0.181896	0.0416	0.711267	0	0.283797
Sh_hnhs	0.266284	0.545479	0.045821	0.16658	0.066483	0.022164	0.095787	0.606825	0.631158	0

Note: F value is in upper-right triangular region and p value is in lower-left triangular region.
